# Efficacy of short-ragweed sublingual immunotherapy tablet MK-3641 in monosensitized and polysensitized subjects

**DOI:** 10.1186/1710-1492-10-S2-A31

**Published:** 2014-12-18

**Authors:** David I Bernstein, Kevin R Murphy, Hendrik Nolte, Amarjot Kaur, Jennifer Maloney

**Affiliations:** 1 Bernstein Allergy Group, Cincinnati, OH, USA; 2 Boys Town National Research Hospital, Boys Town, NE, USA; 3 Merck & Co., Whitehouse Station, NJ, USA

## Background

Immunotherapy for allergic rhinitis with/without conjunctivitis (AR/C) may exhibit different efficacy characteristics in patients with multiple allergen sensitizations than monosensitized patients. It has been considered that monosensitized patients may benefit more from immunotherapy than polysensitized patients. Evidence from randomized, blinded, placebo-controlled trials of Timothy grass sublingual immunotherapy tablet (SLIT-T) MK-7243 (Merck/ALK-Abelló) indicates that treatment in mono- and polysensitized subjects is equally effective.

## Methods

A prospective efficacy analysis was performed between monosensitized and polysensitized subjects treated with the short-ragweed SLIT-T MK-3641 (*Ambrosia artemisiifolia*; Merck/ALK-Abelló). Pooled data from 2 randomized placebo-controlled trials investigating MK-3641 (6 and 12 Amb a 1-U doses) were used. The primary efficacy outcome was the total combined score (TCS=symptom+medication scores) during the 15-day peak season.

## Results

Differences versus placebo for the MK-3641 6 and 12 Amb a 1-U pooled groups (mono- and polysensitized subjects combined) for the peak season TCS were 20% (−1.70; 95% CI, −2.55 to −0.86) and 23% (−2.02; 95% CI, −2.87 to −1.17), respectively (*P*<0.001 for both). Differences versus placebo in the monosensitized MK-3641 pool (n = 175) were 15% (−1.34; 95% CI, −3.40 to 0.73) and 19% (−1.72; 95% CI, −3.63 to 0.20) for 6 and 12 Amb a 1-U, respectively. In the polysensitized MK-3641 pool (n = 784) difference versus placebo were 21% (−1.78; 95% CI, −2.80 to −0.75) and 27% (−2.27; 95% CI, −3.27 to −1.28) for 6 and 12 Amb a 1-U, respectively.

## Conclusions

In the whole study population, treatment with MK-3641 6 and 12 Amb a 1-U for ragweed-induced AR/C was superior to placebo. Although the sample size for the 2 subpopulations was not balanced and data must be interpreted cautiously, it appears that the treatment effect is similar in the mono- and polysensitized subpopulations, with a numerical trend of a greater treatment effect in polysensitized subjects.

## Trial registration

ClinicalTrials.gov Identifiers: NCT00783198; NCT00770315

